# Referral and Lost to System Rates of Two Newborn Hearing Screening Programs in Saudi Arabia

**DOI:** 10.3390/ijns6030050

**Published:** 2020-06-27

**Authors:** Ahmad A. Alanazi

**Affiliations:** College of Applied Medical Sciences, King Saud bin Abdulaziz University for Health Sciences, Riyadh 11481, Saudi Arabia; alanaziahm@ksau-hs.edu.sa; Tel.: +966-112499999 (ext. 95117)

**Keywords:** hearing screening, hearing loss, lost to system, newborns, referral rate, Saudi Arabia

## Abstract

Congenital hearing loss has been commonly reported as a significant health problem. Lost to system (LTS) is a major challenge facing newborn hearing screening (NHS) programs. This retrospective cross-sectional descriptive study aimed to determine the referral and LTS rates after the two-stage NHS based on transient evoked otoacoustic emissions (TEOAEs) in two main hospitals in Riyadh, Saudi Arabia (SA). NHS was performed on newborns before hospital discharge. Newborns were only rescreened if NHS initially revealed a fail/refer outcome in one or both ears. Those who failed the first and second screenings or had risk factors were referred for auditory brainstem response (ABR) testing to confirm or exclude hearing loss. In total, 20,171 newborns (40,342 ears; 52% males; 48% females) were screened, of whom 19,498 (96.66%) passed the initial screening, while 673 (3.34%) failed. Of the 673 newborns, 235 (34.92%) were LTS, and 438 (65.08%) were rescreened, of whom 269 (61.42%) failed and were referred for a comprehensive audiological assessment to confirm the existence of hearing loss. The referral rate after the initial two-stage screening was equal to 1.33%. The lack of awareness of the importance of NHS among parents seems to be the major cause behind the LTS rate. The stakeholders have to work efficiently to reduce the LTS rate.

## 1. Introduction

Approximately 34 million children around the world have hearing loss [1]. Congenital sensorineural hearing loss affects approximately 2–6 per 1000 newborns in low-, middle-, and high- income countries [2,3]. At least 90% of newborns with hearing loss live in less developed countries [4]. The prevalence of any type and severity of disability among Saudi citizens is 3.3% [5]. According to the General Authority for Statistics in Saudi Arabia (SA), 1.4% of all citizens have mild, moderate or severe hearing difficulties [6]. The prevalence of hearing loss in SA may vary according to the region where the prevalence of hearing loss increases in rural areas [7]. Previous Saudi studies showed different prevalences of hearing loss ranging from 1.75% to 7.12% [8,9]. The General Authority for Statistics in SA reported that 21.3% of disabilities among Saudi citizens are caused by congenital malformations [6]. Consanguineous marriage, which is part of the social customs in SA, leads to many types of the hereditary progressive cochleovestibular anomalies. Thus, syndromic and non-syndromic hereditary hearing loss have been found in many families [10]. 

Most children with congenital hearing loss are potentially identifiable by hearing screening [11]. Therefore, Joint Committee on Infant Hearing (JCIH) urgently recommended to identify hearing loss by one month of age via newborn hearing screening (NHS), diagnose hearing loss by three months of age, and enroll in early intervention by six months of age (i.e., known as the 1–3–6 early hearing detection and intervention [EHDI] timeline) [12]. EHDI programs begin with hearing loss detection, conducted by NHS, which is also called neonatal hearing screening, so diagnosis and intervention can occur at an early age, and the effects of undiagnosed hearing loss on the child, family, and society can be avoided. Without proper identification and intervention, hearing loss hinders children from perceiving speech clearly, which leads to attention and learning difficulties, deprives their language, and delays them from being social competent [13,14,15,16,17,18]. As a result, mental and behavioral problems, risk of failing grades, and expensive special educational cost are expected [19,20,21,22]. The annual worldwide cost of unaddressed hearing loss is approximately estimated at US$ 750 billion including the costs of health sector (excluding the cost of hearing devices), educational and social support, and loss of productivity [1].

Otoacoustic emissions (OAEs) and auditory brainstem response (ABR) are the mostly used technologies for NHS. Pass or fail/refer is displayed on the screen according to the responses recorded from the cochlea or the brainstem [23]. OAEs and AABR screeners do not require behavioral responses from the newborn nor demand interpretation by the examiner. Four available protocols are used in NHS programs: (a) OAEs only, (b) ABR only, (c) OAEs with immediate ABR rescreening if OAE is not passed, or (d) OAEs and ABR [24]. Every protocol has its own strengths and weakness. For example, false positives are less with ABR screening, which has high sensitivity and specificity (i.e., a low referral rate) and is less susceptible to ear canal debris compared to OAE screenings [25]. However, OAE screening, either transient evoked otoacoustic emissions (TEOAEs) or distortion product otoacoustic emissions (DPOAEs), is less expensive and has shorter test time than ABR test time [25,26]. Vohr et al. compared three NHS protocols: TEOAEs, Automated ABR (AABR), and the combination of both. Their results revealed that AABR had higher costs during pre-discharge screening but lower referral rates than either TEOAEs or the combination of both [26]. 

Generally, the diagnosis of infants with hearing loss begins with an appropriate hearing screening protocol [12,27]. The use of tympanometry, OAEs, frequency-specific ABR assessments is recommended to diagnose hearing status in infants from birth to six months of age [12,27]. By the age of six months, behavioral hearing thresholds are used to confirm the diagnosis of hearing loss. That said, ABR threshold estimates have been initially used to predict behavioral hearing thresholds within 5–20 dB and program hearing aids [28,29]. NHS is cost effective, and “a two-stage screening, including scheduling and tracking the babies into a diagnostic evaluation, but not including the cost of the diagnosis itself, was approximately US$ 26 per baby” [22]. Some studies have found that the mean age of identification of hearing impairment was 12–13 months before NHS programs [30]. However, with advances in technology and improvement of NHS protocols, many infants with hearing loss are identified at a few weeks of age, and the age of diagnosis has significantly reduced to 2–3 months [31,32,33,34]. The US Centers for Disease Control and Prevention (CDC) reported that the percentage of newborns screened for hearing loss shortly after birth in 50 states and seven territories was 98.3% (Range = 88.9–99.9%) [35]. Although NHS programs were implemented in developed countries as standard practice mandated by governments, several less developed countries have not mandated such programs [2,36].

In SA, where public and private healthcare sectors exist, hearing screening, diagnosis, and intervention are provided for all eligible individuals at no charge in almost all Ministry of Health (MOH) and other stakeholders’ (e.g., Ministry of National Guard and Ministry of Defense) hospitals and medical cities. Furthermore, health insurance companies cover the cost of NHS and other re/habilitative services (e.g., hearing aids up to approximately US$ 2700) for beneficiaries who are treated in private hospitals [37,38]. Still, NHS programs are not standardized in SA. For instance, no unified NHS (e.g., different NHS protocols) exists between public and private healthcare facilities. The initial efforts to standardize NHS programs began in two hospitals, located in Riyadh, as a collaboration between the MOH and Saudi Association for Hearing Impairment in 2007.

A few years later, the Saudi government legislated the national NHS and early intervention of hearing impairment program and the national registry of hearing impairment cases in 2014 as part of the national newborn screening program for metabolism and endocrine disorders [39]. The MOH has launched the first phase of the NHS program that covered more than 60% of newborns in 30 referral hospitals in 2016 and aimed to screen all newborns within 72 h of delivery [40]. Of the screened newborns, 75% had their audiological evaluation finalized by 4–5 weeks of age [7]. The MOH also designed a unified database and e-system for registering screening results and monitoring quality and performance through periodical reports [40]. Consequently, the number of newborns screened for hearing loss has increased from 17% to 89% [41]. American Speech-Language-Hearing Association (ASHA) suggested that the benchmark for the percentage of newborns who complete inpatient and outpatient hearing screening is 95%, while 90% is the benchmark for newborns who did not pass the screening and were referred for diagnosis by three months of age [42]. 

Despite the improvement in access to NHS programs, lost to follow-up (LTF) and lost to documentation (LTD) are still major obstacles. LTF means that the baby did not receive or complete the recommended diagnostic or intervention process [43]. Lost to documentation (LTD) means that “infants who did not pass their hearing screening and whose diagnostic or intervention status has not been reported to the EHDI program; thus, their status remains unknown by the EHDI program despite the fact that they may have received services” [44]. To reduce confusion, lost to system (LTS), a broad heading that combines both LTF and LTD and suggested by ASHA, will be used to represent both terms in the current study [44]. According to the CDC, the overall LTS rate for screening was 0.6% (Range= 0.0–8.4%), whereas the overall LTS rate for diagnosis was 24.6% (Range= 0.0–86.7%) [35]. LTS is a result of several challenges that impact the effectiveness and efficiency of NHS programs in both developed and less developed countries, such as cost, geographical location because of the shortage of well-trained professionals, parental education level (parents’ literacy), and healthcare professionals’ knowledge and team collaboration [45,46,47,48,49]. 

Cost is not a pertinent cause for missing follow-up appointments in SA, because the services are either provided free of charge or covered by health insurances. However, the time of hearing loss detection among Saudi children is affected by the geographical location [45]. The limited services and lack of well-trained audiologists and speech-language pathologists in some Saudi regions may delay early identification of hearing loss and consequently postpone access to intervention and adequate services [7]. The mean age of cochlear implantation in SA is 45.7 months compared to 21.5 months in the United States [45].

Less than 10% of newborns do not pass the screening prior to discharge, and are referred for further diagnosis, so parents’ knowledge of the importance of the EHDI timeline is crucial to avoid missing the confirmatory testing and intervention [22,35]. Their lack of awareness of the EHDI timeline leads to poor follow-up return rate [12,31,48]. A few awareness programs about the importance of hearing screening and the negative consequences of hearing loss exist in SA, most of which are organized by hospitals as individual initiatives with limited access to all public. Parents should be educated about the importance of hearing in development and the serious consequences of hearing loss in their baby’s life [22]. Additionally, healthcare specialists also have much misinformation regarding the ability to test infant hearing and the importance of NHS and follow-up appointments [50]. Those providers did not consider family education about NHS to be a priority [51]. This misunderstanding could be more apparent when there is insufficient equipment, weak training, and scarcity of qualified personnel [52].

Although the challenges that lead to LTS, which, in turn, impact NHS programs may be reasonably anticipated in SA, no study has explored the LTS rate of NHS programs. The need for a study that provides new data about the referral and LTS rates is significant. Research in this area is noteworthy for improving screening outcomes and making recommendations. Therefore, this study aimed to measure the referral and LTS rates after the initial NHS based on TEOAEs in two main Saudi hospitals.

## 2. Materials and Methods

This retrospective descriptive, cross-sectional study was conducted in two specialized maternity departments located in King Saud Medical City and Al Yamamah hospital in Riyadh. This study was approved by King Abdullah International Medical Research Center Institutional Review Board under protocol #RC19/373/R (approved on 26 September 2019). All available NHS results restored in the computerized database in both hospitals for three years were used. No standard statistical analysis procedures were used to estimate frequencies and proportions for categorical variables, such as maternal age and education. Instead, descriptive statistics was used to analyze data. 

### 2.1. Study Population 

Hearing screening with TEOAEs was performed before hospital discharge for 20,171 newborns (40,342 ears). Newborns were screened at several points in time according to the time of delivery. The screening was mainly done within two days after giving birth. There were 10,496 (52%) males and 9675 (48%) females. All newborns’ families were from Riyadh city or neighboring suburbs.

### 2.2. Screening Protocol

The “OAEs only” protocol was used in both study settings. This protocol stated that every newborn has to be screened before discharge. All mothers of newborns were provided with general information about the NHS program by trained nurses prior to screening. Every newborn was screened with TEOAEs. Both ears were screened individually. The screening was conducted in a special room designed for this purpose within the maternity departments. If one or both of the newborn’s ears failed/referred the first screening test, the second hearing screening was conducted before the mother discharged from the hospital. However, mothers, in most cases, were discharged from the hospital within two days after giving birth; therefore, the second hearing screening could not be performed. Parents were informed to bring their baby for another hearing screening within two weeks. 

If one or both of the newborn’s ears did not pass the second hearing screening or if the newborn was at risk of developing progressive or late onset hearing loss, then he/she was referred for ABR testing no later than three months after the second screening. All TEOAE screenings were performed by trained nurses and supervised by a licensed audiologist. The newborns’ demographic information, screening results and risk factors were documented in a special form and then inputted manually in the computerized database immediately after each screening or later at the same day of the screening. Recently, the AABR protocol has been used in all NHS programs supported by the MOH [40,41]. 

### 2.3. Instrumentation 

The screening techniques provided a pass or fail/refer result without the need for a subjective data analysis. TEOAE screenings were conducted using the GSI AudioScreener. Two screeners were used in each hospital. The device used in-ear calibration before screening commenced. The probe of the device was placed in the external ear canal of every newborn with a rubber tip. The collection parameters were as follows: the stimulus type was click; the click rate was 50–80 per second; the stimulus intensity was 80–85 dB pe SPL, and the frequency range was 1000–5000 Hz testing five frequencies. The screening can detect bilateral or unilateral hearing loss in this frequency range. To achieve the “pass” criteria, the newborn must pass four out of five frequencies screened in each ear with the presence of a response as a signal-to-noise ratio of at least six dB (or an overall minimum amplitude response of 6 dB) and a reproducibility of ≥ 50%. 

## 3. Results

**Figure 1:**
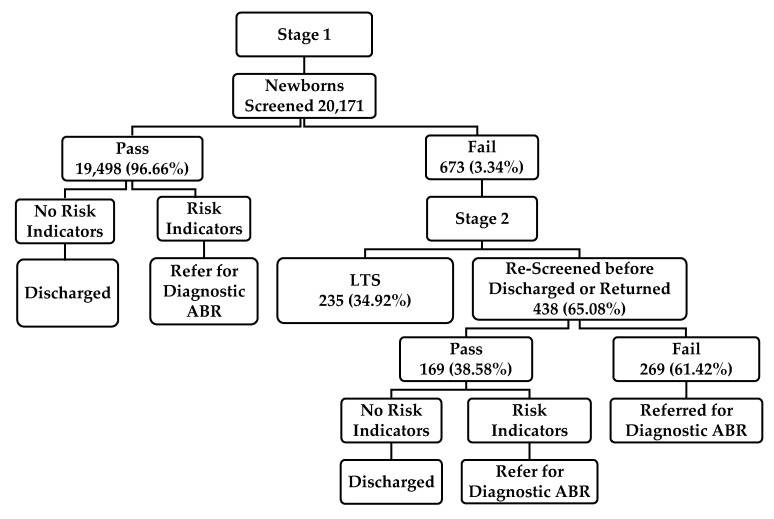
The pass and referral rates of the two-stage NHS protocol used in two Saudi hospitals. Note: LTS: Lost to system; ABR: Auditory brainstem response.

A total of 20,171 newborns were screened in both hospitals (10,496 males; 9675 females). The initial TEOAEs pass rate was 96.66% (19,498 newborns). Of the 673 (3.34%; 351 males; 322 females) newborns who failed the first-stage screen, 438 (65%) were screened before hospital discharge or within two weeks in the second-stage screen. Of the 438 newborns, 169 (38.58%) passed the TEOAE measures, and 269 (61.42%) failed and were referred for ABR testing (Figure 1). This study revealed that the referral rate after the initial two-stage screening was equal to 1.33%. The exact rate might be more or less if the LTS cases and confirmed hearing loss among referred cases for ABR testing were included. Unexpectedly, 235 out 673 (34.92%) newborns were LTS. The number of newborns who were in the regular nursery, the number of unilateral or bilateral failed cases, the ABR results of those who failed the two-stage screening, and the LTS rate after the second stage could not be verified according to the limited available data in the computerized database.

## 4. Discussion

NHS is one part of a comprehensive EHDI program of service. NHS programs have been implemented throughout the world with a primary goal of early identification of newborns who are likely to have hearing loss and who require additional evaluation. LTS minimizes the benefits of these programs. The current study investigated the referral and LTS rates of two Saudi NHS programs. Of 20,171 newborns screened, 19,498 newborns passed the first-stage screening and 673 failed, of whom 269 newborns impaired the two-stage TEOAEs. The referral rate after the initial two-stage screening was equal to 1.33%. This rate approximately correlates with the referral rate (1.7%) after the final/most recent screening reported by the CDC [35]. Habib and Abdelgaffar estimated the incidence of congenital hearing impairment in Saudi population around 0.17–0.18% [53]. Al-Shaikh and Zakzouk reported the overall prevalence of sensorineural hearing loss in Saudi Children to be 1.5% [54]. According to the World Health Organization, the prevalence of disabling hearing loss among children in the Middle East and North Africa was 0.9% [55]. NHS protocols used in these studies were similar to the protocol utilized in the current study setting. TEOAEs were used for the two-stage screening, and newborns who did not meet the pass criteria after the second step were referred for comprehensive audiologic assessments to confirm the presence of hearing loss. This is consistent with the recommendations proposed by JCIH [12]. 

The LTS rate (34.92%), found in this study, was extremely higher than the overall LTS rate for screening, 0.6%, reported by the CDC [35]. Several causes for poor follow-up return rate were reported in the literature. Although the geographical location does not seem to be a pertinent factor for LTS in the current study because parents of the screened newborns lived in Riyadh or neighboring suburbs, this factor may play a vital role in other Saudi regions. The lack of awareness of the importance of NHS among parents seems to be the major cause behind the LTS rate compared to other obstacles discussed in the introduction section. The success of children with hearing loss is affected by parent’s education, attitudes (e.g., reactions and acceptance) and encouragement for their child [56]. Al-Sayed and AlSanosi reported a great waste of time between the first observation of the impairment by the family and the time of cochlear implantation in SA [41].

Unfortunately, limited number of awareness campaigns about the importance of NHS programs and the negative consequences of hearing loss on the individual’s development exist in SA. Such campaigns are considered crucial in health promotion and hearing loss prevention and expected to enhance parents’ (and the population) literacy and consequently encourage them to adhere to the NHS programs. Indeed, the MOH and other stakeholders need to support those campaigns to promote health against health risks, which is one of the strategic objectives of the National Transformation Program aimed to realize Saudi Vision 2030 [57].

Parents should be well informed and included in EHDI programs to help them make knowledgeable decisions and examine all options if their child is identified with hearing loss [42]. The benchmark for the percentage of infants who obtain amplification within one month of hearing loss confirmation is 95% [42]. That said, having a newborn identified with hearing loss is a difficult and challenging experience for most parents. It is estimated that only 22% of parents recognize what the next step is when their child is identified with hearing loss [49]. Therefore, audiologists (or nurses in NHS programs) are responsible for helping parents by (a) assisting them to understand the results, (b) giving them a clear cut message about what to do next, (c) giving them multiple opportunities to hear the same message, (d) providing take-home information, and (e) inviting them to call back with questions, because it is difficult for parents to process and understand counselling and recommendations when their emotions are high [58]. Finally, the expansion of EHDI services, not only NHS, in all regions across the country is vital. All hearing related healthcare professionals should be aware of the 1–3–6 EHDI timeline and the technology used to screen infant hearing, which emphasizes the need of interprofessional education and practice.

## 5. Limitations 

The study had some limitations. Although the estimation of hearing loss prevalence was not the aim of the study, it needs to be quantified based on the audiological confirmation (e.g., the results of the diagnostic ABR test). The most definitive determination of the prevalence of hearing loss are achieved by using (a) gold-standard test data via behavioral audiometry when available and (b) longitudinally when all other children missed by the screening program are identified at a later age. The database used to extract information provided limited data about the number of newborns who were at risk, the number of unilateral or bilateral failed cases, and the ABR testing results for those who failed the two-stage screening. The manual entry of the NHS results to the database could be the reason behind some of the missing information. Although some possible predictors for the LTS rate were reported, the investigation would have more depth if reasons for LTS were surveyed. The protocol used TEOAEs to test frequencies above 4000 Hz, so stimulus artefact might have affected the test reliability of these frequencies. Furthermore, TEOAEs are less sensitive to auditory neuropathy, a neural hearing loss that leaves cochlear outer hair cells’ function intact, compared to ABR. Thus, auditory neuropathy cases may have been missed.

## 6. Recommendations and Future Research 

To minimize the LTS rate, the MOH and other stakeholders are recommended to (a) standardize NHS protocols and improve coordination, integrated data management, and tracking systems among all governmental and private hospitals; (b) establish a follow-up center and computer tracking system reach and notify parents a few days before the recommended visit; (c) use automatic transfer of data from the screeners to the database without being manual inputted; (d) train hearing screening personnel on the 1–3–6 EHDI timeline including effective parental counselling; and (e) conduct a national survey to measure the exact LTS rate and factors lead to this dropout. The next step after meeting the 1–3–6 EHDI timeline is to strive for implementing the 1–2–3 EHDI timeline (i.e., identify hearing loss by one month of age via NHS, diagnose hearing loss by two months of age, and enroll in early intervention by three months of age) [59]. Further research is needed to (a) survey the causes for LTS, (b) study the impact of awareness campaigns of the population and consequent adherence to NHS programs, and (c) document newborns who were LTF after the diagnostic stage and those who were confirmed with hearing loss and had no recognized records of early intervention services.

## 7. Conclusions

Early identification of hearing loss via NHS programs paves the way to early confirmation and intervention. The study findings showed that the referral rate of two NHS Saudi programs was almost correlated with the CDC rate; however, the LTS rate was tremendously higher than the LTS rate for screening reported by the CDC. The lack of awareness of the importance of NHS among parents seems to be the major cause behind the LTS rate. Parents need to be well educated about the importance of NHS programs. The results of this study provide the MOH and other stakeholders with valuable evidence about the need for reducing the LTS rate. The percentage of screened newborns in all SA (89%) reported by the MOH needs to match at least the benchmark (95%) suggested by ASHA. Increase early diagnostic evaluation and early intervention services for children in all regions of SA is essential.
